# The effect of preserving prepared sperm samples at room temperature or at 37 °C before intrauterine insemination (IUI) on clinical pregnancy rate

**DOI:** 10.4274/tjod.31644

**Published:** 2015-03-15

**Authors:** Tayfun Çok, Pınar Çağlar Aytaç, Erhan Şimşek, Bülent Haydardedeoğlu, Hakan Kalaycı, Halis Özdemir, Esra Bulgan Kılıçdağ

**Affiliations:** 1 Başkent University Faculty of Medicine, Department of Obstetrics and Gynecology, Ankara, Turkey

**Keywords:** intrauterine insemination, clinical pregnancy rate, sperm preparation, temperature

## Abstract

**Objective:**

The comparison of the effect of preserving prepared sperm samples at room temperature or at 37 °C before intrauterine insemination (IUI) on clinical pregnancy rate.

**Materials and Methods:**

Retrospective clinical research. University hospital, infertility clinic. Patients with one or two follicles, between the ages of 20 and 40, whose infertility period was less than 6 years and the injected total motile sperm count was more than 10 million. Preserving sperm samples prepared for IUI at 37 ºC or at room temperature before IUI.

The clinical pregnancy rate of IUI cycles between 1^st^ of January 2004 and 1^st^ of December 2011 in which prepared sperm samples were preserved at 37 ºC and the clinical pregnancy rate of IUI cycles between 1^st^ of December 2011 and 31^st^ of May 2014 in which prepared sperm samples preserved at room temperature.

**Results:**

Clinical pregnancy rates were similar in IUI cycles in which prepared sperm samples were preserved at 37 ºC and at room temperature (9.3% vs. 8.9%). Clinical pregnancy rates in IUI cycles with 2 follicles were higher than IUI cycles with 1 follicle (10.8% vs. 7.6%) (p=0.002). Further statistical analysis after splitting data according to the number of the follicles revealed that there was no statistical difference between clinical pregnancy rates after IUI cycles in which prepared sperm samples were preserved at 37 ºC or at room temperature in both one follicle (7.6% vs. 7.6%), and two follicle cycles (11.5% vs. 10.1%).

**Conclusions:**

Preserving prepared sperm samples at room temperature had no negative effect on clinical pregnancy rates when compared with reserving prepared sperm samples at 37 ºC during IUI cycles.

## INTRODUCTION

Intrauterine insemination (IUI) is used in combination with controlled ovarian hyperstimulation (COH) in treating couples with unexplained infertility, mild-moderate male factor, stage 1-2 endometriosis, anovulation or cervical factor^(1,2)^. Factors including woman’s age, presence of endometriosis, COH agents, cause of infertility, total motile sperm count (TMSC) given and uterine anomalies have been investigated with respect to their effects on the success of IUI^(3,4,5,6)^. As a changeable and customizable factor, sperm preparation techniques also have the potential of affecting the IUI results. There are many studies exploring the effects of sperm preparation techniques and the temperature of the environment in which sperms are preserved until insemination on the sperm parameters. However, the number of clinical studies investigating the effect of temperature on the success of IUI is limited^(7,8,9,10)^. It has been shown in a study by Petrella et al. that sperms prepared with the gradient method and stored at room temperature retain their motility and vitality for a longer period than sperms stored at 4 °C and 37 °C^(9)^. Until 2011, sperm samples had been prepared for insemination at 37 °C and preserved at 37 °C until insemination. Also in our clinic, sperm samples started to be prepared at 37 °C but preserved at room temperature until insemination from December 2011 onwards.

In this study, we planned to compare the IUI success rates in periods when sperms are preserved at 37 °C versus at room temperature.

## MATERIALS AND METHODS

In this study, we compared the IUI results from January 2004 to December 2011 and the IUI results from December 2011 to May 2014. The first two cycles of patients older than 20 years and younger than 40 years were assessed. Patients who had infertility for more than 6 years and cycles where the TMSC injected was less than 10 million were excluded from the study. Since IUI can be applied to patients with only one or two follicles as per the regulation Assisted Reproductive Treatment Methods issued in March 2010, cycles involving three or more follicles were also excluded from the study to be able to compare similar cycles between the two periods.

### Assessment of Patients

After the patients were assessed through a hormonal analysis and a transvaginal ultrasonography (USG) on the 3^rd^ day of menstruation, hysterosalpingography (HSG) and saline infusion sonography tests were administered between days 6 and 12 of the cycle. Ovulation was assessed with a mid-lutealprogesterone test between days 21 and 26. Following a 3-7 days of sexual abstinence, spermiogram was applied to the men. Patients who had their TMSC more than 5 million but had abnormal sperm parameters according to the World Health Organization were diagnosed with mild-moderate male factor. Couples who had all their tests normal were diagnosed with unexplained infertility. Couples who had oligoanovulation and could not achieve pregnancy although COH-IUI treatment was administered at least in three cycles of ovulation.

### Controlled Ovarian Hyperstimulation

For controlled ovarian hyperstimulation, recombinant follicle-stimulating hormone (FSH) preparations (Puregon, Merck Sharp Dohme, Turkey; Gonal-F, Merck Serono, Turkey), human menopausal gonadotropin (hMG) preparations (Menogon and Menapur, Ferring, Turkey; Merional, IBSA, Turkey), and highly purified urinary FSH preparation (Fostimon, IBSA, Turkey) were started between the 2^nd^ and 4^th^ days of the patient’s menstruation in doses ranging between 50 and 150 IU depending on the patient’s age, the number of antral follicles in her transvaginal USG and her body mass index. For ovulation induction or controlled ovarian hyperstimulation, 50-150 mg of clomiphene citrate (Klomen, Koçak Farma İlaç ve Kimya Sanayi A.Ş.) was started for some patients between the 5^th^ and 9^th^ days of the cycle. we started to monitor the patient on the 6^th^ day of the stimulation during the gonadotropin cycles and the 12^th^ day of the cycle during the clomiphene citrate cycles with transvaginal USG. The frequency of follow-up visits and medicine dosing were adjusted according to the follicle sizes. To trigger ovulation, the presence of at least one follicle of 18 mm and larger was considered sufficient. Cycles involving 3 or more follicles larger than 16 mm were excluded from the study. To this end, we used 5000 IU of urinary human chorionic gonadotropin (hCG) (Pregnyl, Merck Sharp Dohme, Turkey) or 6500 IU of recombinant CG (Ovitrelle, Merck Serono). We made a single IUI 35-40 hours after applying hCG in all cycles.

### Sperm Preparation

Semen samples taken from the patients were kept in a sterile sperm collection container in the 37 °C part of the laminar flow unit (K-Systems, Denmark) for 15-30 minutes for liquefaction. When preparing the semen samples, first 2 ml of 80% gradient medium (Suprasperm, Tek Medikal Servis, Denmark) and then 2 ml of 55% gradient medium (Suprasperm, Tek Medikal Servis, Denmark) were placed in a conical tube (Falcon 2095, Aksuvar ve Asist Medikal, USA) with the help of a 1 ml pipette (Falcon 7521, Aksuvar ve Asist Medikal, USA). These procedures were carried out in the 37 °C part of the unit. The entire semen sample, which completed the liquefaction reaction, was stirred with a 2 ml pipette (Falcon 7507, Aksuvar ve Asist Medikal, USA) and then was counted. Afterwards, the entire sperms were eased on the gradient medium from the tube wall and were centrifuged at 300 g (1200 rpm) (Heraeus Labofuge 400, Japan) for 20 minutes. The centrifuge was not heat controlled. After the centrifugal procedure, the supernatant part was separated and the pellet at the bottom of the tube was put into a round bottom tube (Falcon 2001, Aksuvar ve Asist Medikal, USA) and 3 ml of washing medium (Medicult IVF, Tek Medikal Servis, Denmark) was added on top of it. It was centrifuged once more at 300 g (1200 rpm) for 10 minutes. At the end of the procedure, the supernatant part was separated. Some more washing medium (Medicult IVF, Tek Medikal Servis, Denmark) was added on the pellet to make the final volume of 0.6 ml and it was moved into a 5 ml tube (Falcon 2003, Aksuvar ve Asist Medikal, USA) with a 1 ml pipette (Falcon 7521, Aksuvar ve Asist Medikal, USA) to perform its final count. The prepared sperm samples were then stored in an incubator (Heraus Functional Line, Germany) at

37 °C before May 2011 and at room temperature in the unheated part of the laminar flow unit after May 2011.

### Intrauterine Insemination

The prepared semen samples were placed in the intrauterine cavity with an insemination catheter (Wallace, Smiths Medical International Ltd, UK) 35-40 hours after the ovulation triggering with hCG in the patients. Following a 10-minute rest, the patients were advised to return to their routine activities. When 14 days have passed after IUI, a β-hCG test was carried out for patients who did not have menstruation. The patients with a positive result were assessed with a transvaginal USG two weeks later and those who had fetal heart beat were considered as being pregnant.

### Statistical Method

The SPSS for Windows 17.0 (SPSS Inc. Chicago, IL, USA) was used for statistical analyses. Percentages and frequencies were used for categorical data and means and standard deviations for continuous data. The Kolmogorov-Smirnov test was run for normal distribution analyses. The student’s t-test was used for intergroup differences if there was a normal distribution and the Mann-Whitney U-test, if there was not a normal distribution. The chi-square test or, when necessary, the Fisher’s exact test was run to analyze the pregnancy rates in the IUI cycles. The Pearson correlation test was applied to explore the correlation between the variables and pregnancy. P values less than 0.05 were considered statistically significant.

## RESULTS

The IUI results from January 2004 to May 2011 and the IUI results from May 2011 to May 2014 were compared in this study. The 7552 cycles of 3812 patients were investigated. In the correlation analysis of the 7552 cycles, the parameters affecting the pregnancy rates were found as age, duration of infertility, basal FSH, number of follicles, number of cycles and TMSC that were inseminated. In order to prevent the effect of these factors and a possible bias, the first two cycles of the patients aged between 20 and 40 years, who had less than three follicles, had infertility for less than 6 years, and had more than 10 million of a total number of motile sperms injected, were included in the study. 3217 cycles that matched these criteria were analyzed.

There was no statistically significant difference between the groups with respect to age, duration of infertility, basal FSH, number of cycles and TMSC that were inseminated, but a difference was seen between the groups with respect to number of follicles (Table 1). Storing sperms at 37 °C or at room temperature did not affect the rate of pregnancy, but the number of follicles affected the rate of pregnancy significantly (Table 2). For this reason, the rates of pregnancy were reassessed in both groups against the number of follicles. It was found after this assessment that the sperm storage temperature had no effect on pregnancy (Table 3).

## DISCUSSION

In the present study, we investigated retrospectively the effects of storing sperms that were prepared with the gradient method at 37 °C and at room temperature on the pregnancy rates in the IUI cycles in combination with COH. We found at the end that there was no negative effect of storing sperms at room temperature instead of 3 °C on the pregnancy rates after preparing the sperms in IUI cycles.

In the IUI treatment in combination with COH, the pregnancy rates range is between 8% and 26% and there are many factors influencing the success^(11)^. Different results have been obtained in studies investigating the effect of various temperatures on sperm motility and vitality. In a study by Petrella et al., it was seen that sperms that were prepared with the gradient method and stored at room temperature retained their motility and vitality for a longer period compared to sperms stored at 4 °C or at 37 °C^(9)^.

Although sperm preparation methods have also been investigated, there are a limited number of studies exploring the effects of the temperature to which sperms are exposed when being prepared for the success of IUI^(10,12)^. In a study by Küçük et al., a statistically significant increase was found in TMSC and pregnancy rates when the sperms were stored at 40 °C for 2 hours^(12)^. It was shown in another study that carrying out the centrifuge procedure at 37 °C or without any heat control did not have any effect on the pregnancy rates^(10)^. In a study showing that the increase in sperm DNA fragmentation affects the pregnancy rates in the IUI cycles negatively, it was found that no pregnancy could be achieved in patients who had more than 12% sperm DNA fragmentation^(13)^. Matsuura et al. have shown that as the waiting time is prolonged, the DNA fragmentation increases in sperms and this increase is more at 37 °C than at room temperature^(14)^. In another study, no statistically significant difference was found when the DNA fragmentation rates in the sperms prepared at 37 °C and at room temperature were compared^(7)^. Contrary to these studies, Franken et al. observed in their study that more motile sperms could be obtained when they were prepared at 34 °C as compared to sperms prepared at room temperature^(8)^. It was shown in another study that sperm capacitation necessary for sperms to inseminate the oocyte did not occur in the sperms stored at 20 °C, but when these sperms were exposed to 37 °C later, their capacitating reactions began^(15)^. In a study by Si, it was found that a temperature value of 37 °C was needed in for hyperactive movements to begin and continue in immotile sperms obtained from hamster epididymis that were exposed to temperatures between 22 and 40 °C^(16)^. We did not find any difference between the motilities of sperms stored at room temperature and at 37 °C. No difference was found between the storage of sperms prepared for insemination at 37 °C and at room temperature in terms of pregnancy rates.

The sperm preparation and storage stage are the steps that can affect the outcome of the IUI treatment^(10)^. Simulating the body temperature or using the environment temperature at this stage may exhibit differences regarding the effort of the technical team and the required equipment. When the evidence-based medicine practices together with a sound resource management, which are important for the contemporary healthcare services, are taken into consideration, the use of low energy and practicality should be the preference at a human or equipment level in every intervention. By showing in the present study that after preparing sperms in the IUI cycles, storing them at room temperature rather than at 37 °C did not have any negative effect on the pregnancy rates, we concluded that storing sperms at room temperature, through which the appropriate conditions are attained more easily, was sufficient to sustain the success of an IUI.

## Figures and Tables

**Table 1:**
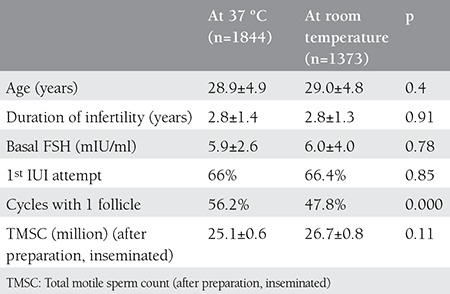
Demographic data

**Table 2:**
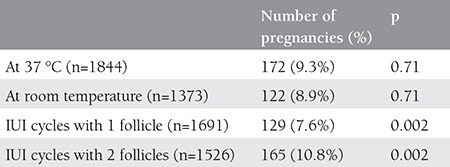
Pregnancy rates with respect to the temperature to which sperms are exposed and the number of follicles

**Table 3:**
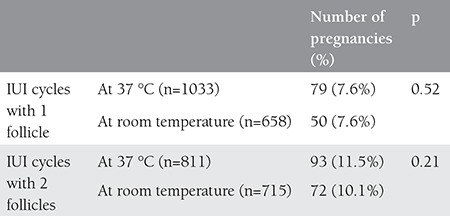
Pregnancy rates with respect to the temperature that sperms are exposed to when the number of follicles is fixed
